# Imaging‐proteomic analysis for prediction of neoadjuvant chemotherapy responses in patients with breast cancer

**DOI:** 10.1002/cam4.6704

**Published:** 2023-11-14

**Authors:** Jingxian Duan, Yuanshen Zhao, Qiuchang Sun, Dong Liang, Zaiyi Liu, Xin Chen, Zhi‐Cheng Li

**Affiliations:** ^1^ Institute of Biomedical and Health Engineering Shenzhen Institute of Advanced Technology, Chinese Academy of Sciences Shenzhen China; ^2^ The Key Laboratory of Biomedical Imaging Science and System, Chinese Academy of Sciences Shenzhen China; ^3^ National Innovation Center for Advanced Medical Devices Shenzhen China; ^4^ Shenzhen United Imaging Research Institute of Innovative Medical Equipment Shenzhen China; ^5^ Department of Radiology Guangdong Provincial People's Hospital, Guangdong Academy of Medical Sciences Guangzhou China; ^6^ Guangdong Provincial Key Laboratory of Artificial Intelligence in Medical Image Analysis and Application Guangdong Provincial People's Hospital, Guangdong Academy of Medical Sciences Guangzhou China; ^7^ Department of Radiology, Guangzhou First People's Hospital, School of Medicine South China University of Technology Guangzhou China

**Keywords:** breast cancer, deep learning, neoadjuvant chemotherapy, pathologic complete response, Radiogenomics

## Abstract

**Background:**

Optimizing patient selection for neoadjuvant chemotherapy in patients with breast cancer remains an unmet clinical need. Quantitative features from medical imaging were reported to be predictive of treatment responses. However, the biologic meaning of these latent features is poorly understood, preventing the clinical use of such noninvasive imaging markers. The study aimed to develop a deep learning signature (DLS) from pretreatment magnetic resonance imaging (MRI) for predicting responses to neoadjuvant chemotherapy in patients with breast cancer and to further investigate the biologic meaning of the DLS by identifying its underlying pathways using paired MRI and proteomic sequencing data.

**Methods:**

MRI‐based DLS was constructed (radiogenomic training dataset, *n* = 105) and validated (radiogenomic validation dataset, *n* = 26) for the prediction of pathologic complete response (pCR) to neoadjuvant chemotherapy. Proteomic sequencing revealed biological functions facilitating pCR (*n* = 139). Their associations with DLS were uncovered by radiogenomic analysis.

**Results:**

The DLS achieved a prediction accuracy of 0.923 with an AUC of 0.958, higher than the performance of the model trained by transfer learning. Cellular membrane formation, endocytosis, insulin‐like growth factor binding, protein localization to membranes, and cytoskeleton‐dependent trafficking were differentially regulated in patients showing pCR. Oncogenic signaling pathways, features correlated with human phenotypes, and features correlated with general biological processes were significantly correlated with DLS in both training and validation dataset (*p*.adj < 0.05).

**Conclusions:**

Our study offers a biologically interpretable DLS for the prediction of pCR to neoadjuvant chemotherapy in patients with breast cancer, which may guide personalized medication.

## 1 INTRODUCTION

Female breast cancer is the most commonly diagnosed cancer worldwide, accounting for 11.7% of total incidences.^1^ Neoadjuvant chemotherapy has become a standard treatment for breast cancer to downsize the tumors before surgical resection.^2^ It was shown to increase breast‐conserving rate, provide an opportunity to monitor drug response, and improve patient prognosis.^3^, ^4^ However, pathologic complete response (pCR) to neoadjuvant chemotherapy was only observed in 14.7% to 52.9% of the patients, depending on the agent administrated and the molecular feature of the tumor.^5^, ^6^ In nonresponders, neoadjuvant chemotherapy may induce breast cancer metastasis through a tumor microenvironment‐mediated mechanism.^7^ 90% of the patients suffered from adverse effects, including grade 4 leukopenia and neutropenia.^8^ Subsequently, identifying responders of neoadjuvant chemotherapy remains a critical need in breast cancer management.

Biomarkers for neoadjuvant chemotherapy were extensively studied. Genomic signatures,^9^ tumor‐infiltrating lymphocytes,^10^ DNA repair deficiency,^11^ and cell checkpoint proteins^12^ were proposed to predict clinical outcomes following neoadjuvant chemotherapy. These next‐generation sequencing‐based methods require invasive tissue sampling, which may induce post‐biopsy breast infection and bleeding. Moreover, the predictive power of these molecular biomarkers highly depends on the molecular subtype of tumors.^13^ A genomic signature was shown to predict response to neoadjuvant chemotherapy in triple‐negative breast cancer with an AUC of 0.75.^14^ On the other hand, machine learning models were established to predict pCR to neoadjuvant chemotherapy based on multiparametric MRIs.^15^, ^16^, ^17^ These noninvasive approaches achieved the prediction AUC (area under curve) of 0.80–0.86 irrespective of tumor subtype. Notably, most machine learning models were built with handcraft radiomic features, which may limit the prediction power by human's understanding of medical imaging.^18^ Deep learning, especially convolutional neural network (CNN) techniques, can improve this handcraft pipeline by automatically learning discriminative features directly from imaging. Developing a CNN‐based imaging signature for prediction of neoadjuvant chemotherapy response has indubitable clinical benefit.

However, the data‐driven nature of deep learning algorithm makes it a black box and thusly hindered its applications in clinical practice. A few pioneer studies managed to map radiomic features to transcriptomic expression patterns and biological functions,^19^, ^20^, ^21^ offering a biologically interpretable artificial intelligence (AI) algorithm for the prediction of patient survival. Notably, most existing radiogenomic studies were based on transcriptomic analysis, which is the middle step of the transcription‐translation cascade. However, changes at transcriptomic levels may not necessarily transform to functional alterations at proteomic level. Proteins are processed and modified posttranslationally. Protein–protein interactions are the basis of biological functions.^22^ Proteomic studies adapted a series of high‐throughput sequencing technologies to quantify proteins and to reveal the effects of their interactions on biological processes.^23^ Nevertheless, so far few radiogenomic studies have been performed to correlate predictive imaging features with proteomic data. To better understand the biological meaning of deep learning image features, proteomic analysis should be performed to correlate deep learning features with the activity of effector proteins involving in various biological functions.

The study aimed to establish and validate a deep learning signature (DLS) from pretreatment MRI to predict pCR to neoadjuvant chemotherapy in patients with breast cancer and to further elucidate the biological meanings of the DLS by revealing biological pathways associated with the DLS using paired MRI and proteomic sequencing data.

## 2 MATERIALS AND METHODS

### 2.1 Study design

**FIGURE 1:**
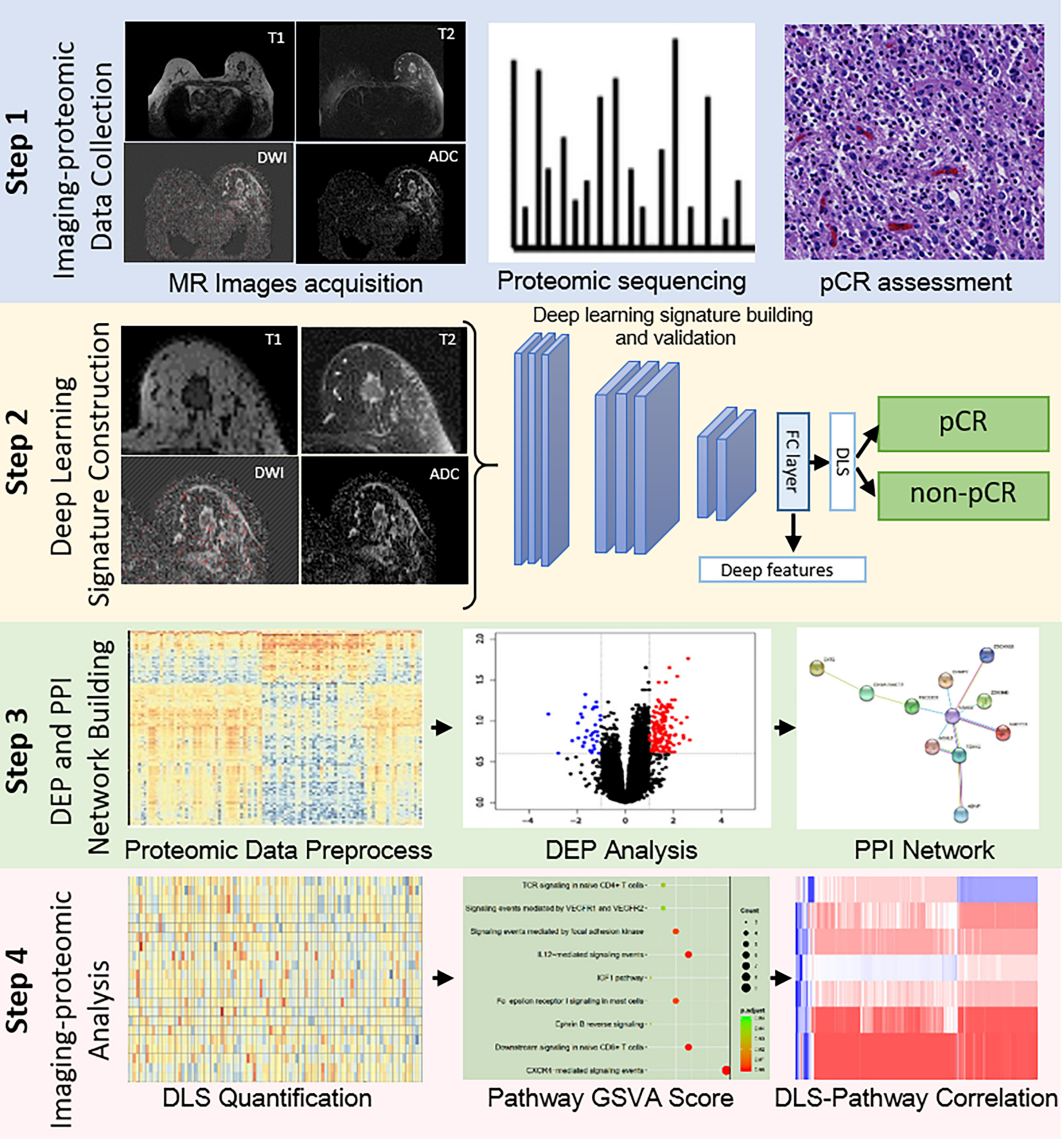
The overview of the study design. The major steps include MR image acquisition, establishment of deep learning signature (DLS) for the prediction of pathological complete response (pCR) to neoadjuvant chemotherapy, proteomic sequencing for identifying deferentially expressed proteins (DEPs) and protein–protein interaction (PPI) network building, and imaging‐proteomic analysis for revealing the biological meanings of the DLS.

This retrospective study comprised four major steps: imaging‐proteomic data collection, DLS construction for predicting pCR using imaging data, identification of molecular features associated with pCR using proteomics data, and investigation of biological pathways underlying the DLS using paired imaging and proteomic data (Figure 1). First, histologically confirmed breast cancer patients who underwent neoadjuvant chemotherapy were enrolled, where pretreatment MRI, proteomic sequencing data, and clinical data were collected. Second, an imaging signature, the DLS, was constructed from pretreatment MRI using a deep learning model for predicting the responses to neoadjuvant chemotherapy. Third, the tumor tissues were sampled by biopsy before neoadjuvant therapy and were sent for proteomic sequencing. Molecular pathways associated with response to neoadjuvant chemotherapy were identified by building protein–protein interaction (PPI) networks using the proteomic sequencing data. Lastly, molecular pathways associated with the DLS were investigated to reveal the biological meaning of the DLS by using the paired MRI and proteomics data.

### 2.2 Patient and dataset

**FIGURE 2:**
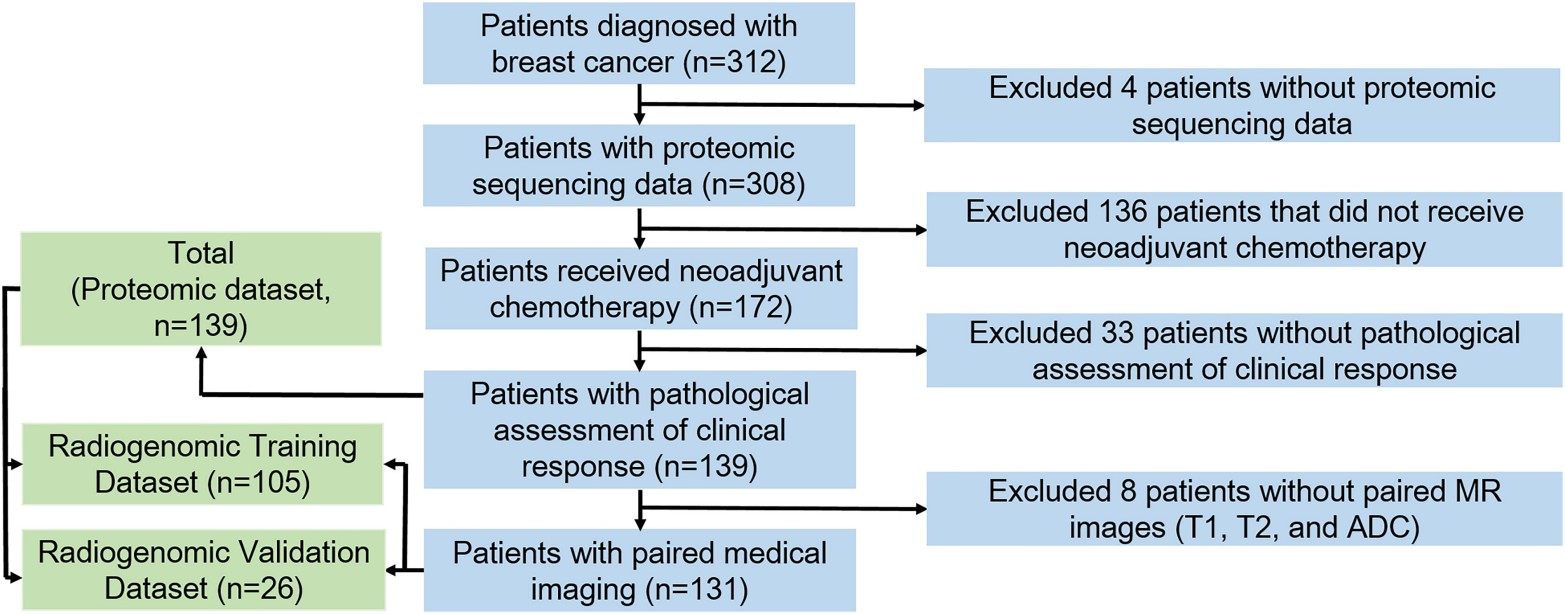
Patient enrollment process of the study cohort. The patient enrollment process and the exclusion criteria were shown in the flow chart.

This study was approved by the Medical Ethics Committee of Guangdong Provincial People's Hospital (GPPH). One hundred thirty‐nine patients with histologically confirmed breast cancer were enrolled at GPPH from March 2016 to August 2019. Patient enrollment process is shown in Figure 2. Core needle biopsy was performed under imaging guidance in concordance with the National Comprehensive Cancer Network (NCCN) guideline,^24^ sampling the tumor cores for each patient. Pretreatment tumor tissue samples extracted by biopsy were sent for proteomic sequencing. All patients received four cycles, six cycles, or eight cycles of neoadjuvant chemotherapy prior to breast surgery following the NCCN guideline.^2^ The neoadjuvant chemotherapy regimens were either taxane‐based, anthracycline‐based, or anthracycline and taxane‐based.

Standard histopathologic analysis was conducted on posttreatment specimens for the pathologic assessment of response to neoadjuvant chemotherapy. pCR was defined as the absence of residual invasive carcinoma in the specimen (residual ductal carcinoma in situ could be present) and the absence of lymph node invasion in the ipsilateral sentinel node or lymph nodes removed during axillary dissection (yPT0/isN0). The patients were divided into pCR group and non‐pCR group based on the results of histopathologic analysis. The status of estrogen receptor (ER), progesterone receptor (PR), and HER2 and the Ki67 index were determined by IHC. Tumors with ≥1% of tumor cells with nuclear staining were defined as ER/PR positive. Tumors with immunohistochemistry staining of 3+ or 2+ with in situ hybridization amplification were defined as HER2 positive.

Paired pretreatment T1‐weighted (T1) MR sequences, T2‐weighted (T2) MR sequences, diffusion‐weighted image (DWI), and the corresponding apparent diffusion coefficient (ADC) map were obtained for 131 patients. These patients were randomly split into the radiogenomic training dataset and radiogenomic validation dataset, comprising 105 and 26 patients, respectively. Written informed consent was obtained for each patient enrolled in this study.

### 2.3 MR image acquisition and preprocessing

Pretreatment T1, T2, and DWI MR images were captured with 3.0 T United Imaging (uMR790, Shanghai, China) MR scanners and 1.5 T GE Medical Systems (Optima MR360, Milwaukee) MR scanners. Axial DWI images were obtained using the *b*‐value of 1000 s/mm^2^ and transformed into ADC maps. T1, T2, and ADC images were preprocessed to normalize intensity and sampling sizes. First, N4ITK was applied to correct bias field distortion. The MR images were rigidly registered using axial resampled T2 as a template. The three‐dimensional volumes of interest (VOI) of tumor contours that contained the largest tumor area were outlined on 6 layers of T2 MR images. T1 and ADC images were used to cross‐check that the extension of the whole tumor area was included. Next, all voxels were isotropically resampled into 1 × 1 × 1 mm^3^ with linear interpolation for consistent feature extraction. A 3D bounding box was derived based on the delineated contours for each patient.

### 2.4 Proteomic sequencing

Pretreatment tumor samples were extracted by biopsy. The sample was transferred to a 1.5‐mL centrifuge tube and lysed with SDT lysis buffer (4% SDS, 10 mM DTT, 100 mM TEAB), followed by 5 min of ultrasonication on ice. The lysate was centrifuged at 12000 *g* for 15 min at 4°C, and the supernatant was reduced with 10 mM DTT for 1 h at 56°C and subsequently alkylated with sufficient iodoacetamide for 1 h at room temperature in the dark. Next, samples were completely mixed with four times volume of precooled acetone by vortexing, incubated at −20°C for at least 2 h, and centrifuged at 12000 *g* for 15 min at 4°C. After washing with 1‐mL cold acetone, the pellet was dissolved by dissolution buffer (8 M urea, 100 mM TEAB, pH 8.5).

Proteins were digested by trypsin and CaCl2. Formic acid was mixed with digested sample, adjusted pH under 3, and centrifuged at 12000 *g* for 5 min at room temperature. The supernatant was slowly loaded to the C18 desalting column, washed with washing buffer (0.1% formic acid, 3% acetonitrile) three times, and then added elution buffer (0.1% formic acid, 70% acetonitrile). The eluents of each sample were collected and lyophilized.

Mass spectrometry (MS) was operated under a data‐independent acquisition (DIA) mode. Mobile phases A (0.1% FA in H_2_O) and B (0.1% FA in 80% ACN) were used to develop a gradient elution. A half sample containing 4 μg fraction supernatant and 0.8 μL iRT reagent was injected into the EASY‐nLCTM 1200 UHPLC system (Thermo Fisher) coupled with an Orbitrap Q ExactiveTM series mass spectrometer (Thermo Fisher) operating in the data‐independent acquisition mode with spray voltage of 2.1 kV, Nanospray Flex™ (ESI), and capillary temperature of 320°C. For DIA acquisition, the m/z range covered from 350 to 1500. MS1 resolution was set to 60,000 (at m/z 200), full scan AGC target value was 5 × 105, and the maximum ion injection time was 20 ms. Peptides were fragmented by HCD in MS2, in which resolution was set to 30,000 (at 200 m/z), and AGC target value was 1 × 106, a normalized collision energy of 27%.

The resulting spectra from each fraction were searched by Proteome Discoverer 2.2 (PD 2.2, Thermo). The result of search and identification by PD2.2 software was imported into Spectronaut (version 14.0, Biognosys) software to generate a library.^25^ Ion‐pair chromatographic peaks were extracted according to target list. Matching the ion and calculating peak area to achieve the qualitative and quantitative of peptides.

### 2.5 Deep learning signature building and validation

A deep convolutional neural network model was employed to predict the clinical outcome of neoadjuvant chemotherapy. ResNet‐34 architecture was used as the network backbone. In total, 18 resampled MR slices from T1, T2, and ADC tumor contours were integrated into a 3D tumor profile for each patient. All slices of the 3D tumor profile were resized to 256 × 256 and were fed into the network as input. The network was trained from scratch on the radiogenomic training dataset (*n* = 105) and was optimized on the radiogenomic validation dataset (*n* = 26). Adam optimizer was applied with a learning rate of 10^−5^ and a batch size of 6. Random rotation, shear, and zoom approaches were performed on the preprocessed and cropped images for data augmentation. The output of the network was a probability vector regarding the pCR status. The binary outcome (pCR = 1, non‐pCR = 0) was used as the training labels. The loss function for this model was the cross‐entropy loss, which was written as

(1) $$\text{loss}=\frac{1}{n}\sum_{i}^{1}-\left[y_{i}\log\left(p_{i}\right)+\left(1-y_{i}\right)\log\left(1-p_{i}\right)\right]$$

where *loss* was the loss value of the deep CNN; i was the index of patient; y_i_ and p_i_ were the predicted label and the corresponding probability score of patient i, which were the outputs of the network that predict clinical outcome of patients who received neoadjuvant chemotherapy. The network output was used as the deep learning signature. The network was implemented using the PyTorch (version 1.4.0) library.

### 2.6 Transfer learning

Similar to the above, a pretrained ResNet‐34 model was used as the backbone for the deep learning model. The pretrained model weights were downloaded from https://github.com/pytorch/. The last layer of the ResNet‐34 model was replaced by a fully connected layer with a sigmoid activation function to predict treatment response. The model was fine‐tuned on the training set using the binary cross‐entropy loss function and the Adam optimizer with a learning rate of 10^−5^ and a batch size of 6.

### 2.7 Identification of deferentially expressed proteins and functional enrichment analysis

To reveal the biological functions facilitating response to neoadjuvant chemotherapy, deferentially expressed proteins (DEPs) were enriched to identify key biological pathways that were differentially regulated in the pCR group. Differential expression analysis of the pCR and non‐pCR group was performed using the edgeR package (version 3.34.1).^26^ The resulting *p* values were adjusted using the Benjamini and Hochberg's approach to control the false discovery rate (FDR). Genes with log2 (FoldChange) >1 and *p* value < 0.05 were assigned as DEPs. DEPs were enriched to identify key biological pathways that underlie the clinical responsiveness of neoadjuvant chemotherapy. The enrichment analysis was performed using an R package clusterProfiler based on four annotated databases: Gene Ontology (GO), Kyoto Encyclopedia of Genes and Genomes (KEGG), Hallmark, and Reactome.^27^ *p* < 0.05 was considered as significant enrichment.

Protein–protein interaction network was constructed using the Search Tool for the Retrieval of Interacting Genes Database (STRING) and STRINGdb R package (version 2.4.2).^28^ PPI networks were visualized with Cytoscape software, and the minimum required interaction score was set to 0.4.^29^

### 2.8 Identification of biological pathways associated with DLS

Next, we aimed to identify the biological pathways underlying the DLS using the paired MRI and proteomics data from the radiogenomic training dataset. Gene set variation analysis (GSVA) was performed for each enriched pathway to calculate a patient‐specific GSVA score that quantified the pathway activity.^30^ The statistical correlations between DLS and the pathway GSVA scores were assessed by Pearson's correlation coefficiency. *p* Values were adjusted with the Benjamini and Hochberg's approach using the *p*.adjust function in R. Adjusted *p* < 0.05 was considered significant.

To investigate the biological meaning of DLS in depth, deep features composing the DLS were extracted from the fully connected layer of the deep learning network. Correlation between pathway GSVA scores and 512 deep features was assessed by Pearson's correlation coefficiency. Furthermore, principle component analysis (PCA) was conducted on the 512 deep features to reveal the principle components of the DLS. Correlation between pathway GSVA scores and the top three principle components of the DLS was assessed to reveal the biological meaning of the deep features in low‐dimensional representations.

The biological meaning of DLS was also examined at protein level. The patients were divided to low DLS and high DLS group using a DLS cut‐off of 0.5. Expression of DEPs between the low DLS and high DLS group was compared by Mann–Whitney test. *p* Value less than 0.05 was considered statistically significant.

### 2.9 Statistical analysis

All statistical analyses were performed using R (version 4.1.0), Python (version 3.8), or Graphpad Prism (version 9.3.0 for Windows, GraphPad Software). The data were resented as mean ± standard error if not stated otherwise. The normality of each data set was assessed; datasets that passed the normality test were compared with Wilcoxon test, Mann–Whitney test, or *t‐*test depending on the result of the variation test. Datasets that did not display normal distribution and datasets with significant variation were compared by the Wilcoxon test or Mann–Whitney test. *p* Value less than 0.05 was considered statistically significant. The performance of DLS was assessed by receiver operating characteristic curves (ROCs) and AUC. Decision curve analysis (DCA) was performed to show the net clinical benefit of the model.

## 3 RESULTS

### 3.1 Patient demographics

A total of 139 female patients with histologically confirmed breast cancer were enrolled (Table 1). The mean age of the patients was 48.46 ± 1.76 years. 43.92% of these patients were diagnosed with grade II tumors, whereas 51.35% of the patients carried grade III tumors. 41.55%, 32.87%, and 47.55% of the patients were ER, PR, and HER2 positive, respectively. These patients received neoadjuvant chemotherapy, and 51.35% of them showed pCR. Seventeen patients were excluded from the total dataset for missing paired medical images, and the rest of the patients were randomly split to radiogenomic training (*n* = 105) and radiogenomic validation (*n* = 26) datasets. The mean ages of the total, radiogenomic training, and radiogenomic validation datasets had no statistical difference (*p* = 0.58). The radiogenomic training and radiogenomic validation datasets showed similar patient demographics with the total patient statistics, including the percentage of patients with ER+, PR+, HER2+, and Ki67 ≥15%, the percentage of grade II and III patients, and the percentage of patients who responded to neoadjuvant chemotherapy (Data S1).

**TABLE 1:** Patient demographics.

Characteristics	Total (proteomics, *n* = 139)	Radiogenomic training dataset (*n* = 105)	Radiogenomic validation dataset (*n* = 26)	*p* Value
Age (years)	48.46 ± 1.76	50.70 ± 1.01	48.46 ± 1.76	0.58 (P) 0.31 (R)
Histological grade				
II	65 (43.92%)	48 (45.71%)	12 (46.15%)	…
III	76 (51.35%)	54 (51.43%)	11 (42.31%)	…
n/a	7 (4.73%)	3 (2.86%)	3 (11.54%)	
Estrogen receptor (ER)				
+	59 (41.55%)	45 (45.00%)	10 (40.00%)	…
−	89 (58.45%)	60 (55.00%)	16 (60.00%)	…
Progesterone receptor (PR)				
+	47 (32.87%)	34 (33.66%)	9 (36.00%)	…
−	101 (67.13%)	71 (66.34%)	17 (64.00%)	…
HER2				
+	68 (47.55%)	50 (49.02%)	12 (48.00%)	…
−	80 (52.45%)	55 (50.98%)	14 (52.00%)	…
Ki67				
≥15%	140 (95.89%)	98 (94.23%)	25 (96.15%)	…
<15%	8 (4.11%)	7 (5.77%)	1 (4.85%)	…
Clinical response				
pCR	76 (51.35%)	51 (48.57%)	15 (57.69%)	…
Non‐pCR	72 (48.65%)	54 (51.43%)	11 (42.30%)	…

*Note*: Unless otherwise noted, data are numbers of patients, with percentages in parentheses. Age is represented as means ± standard deviations. *p* Value is calculated for the difference in patient characteristics between three datasets (noted as P) or between the training and validation datasets (noted as R). pCR shorts for pathological complete response. Seventeen patients were excluded from the protemic dataset for missing paired medical images; the rest of the patients were randomly splitted to radiogenomic training and validation datasets.

### 3.2 DLS predicted pCR to neoadjuvant chemotherapy

**FIGURE 3:**
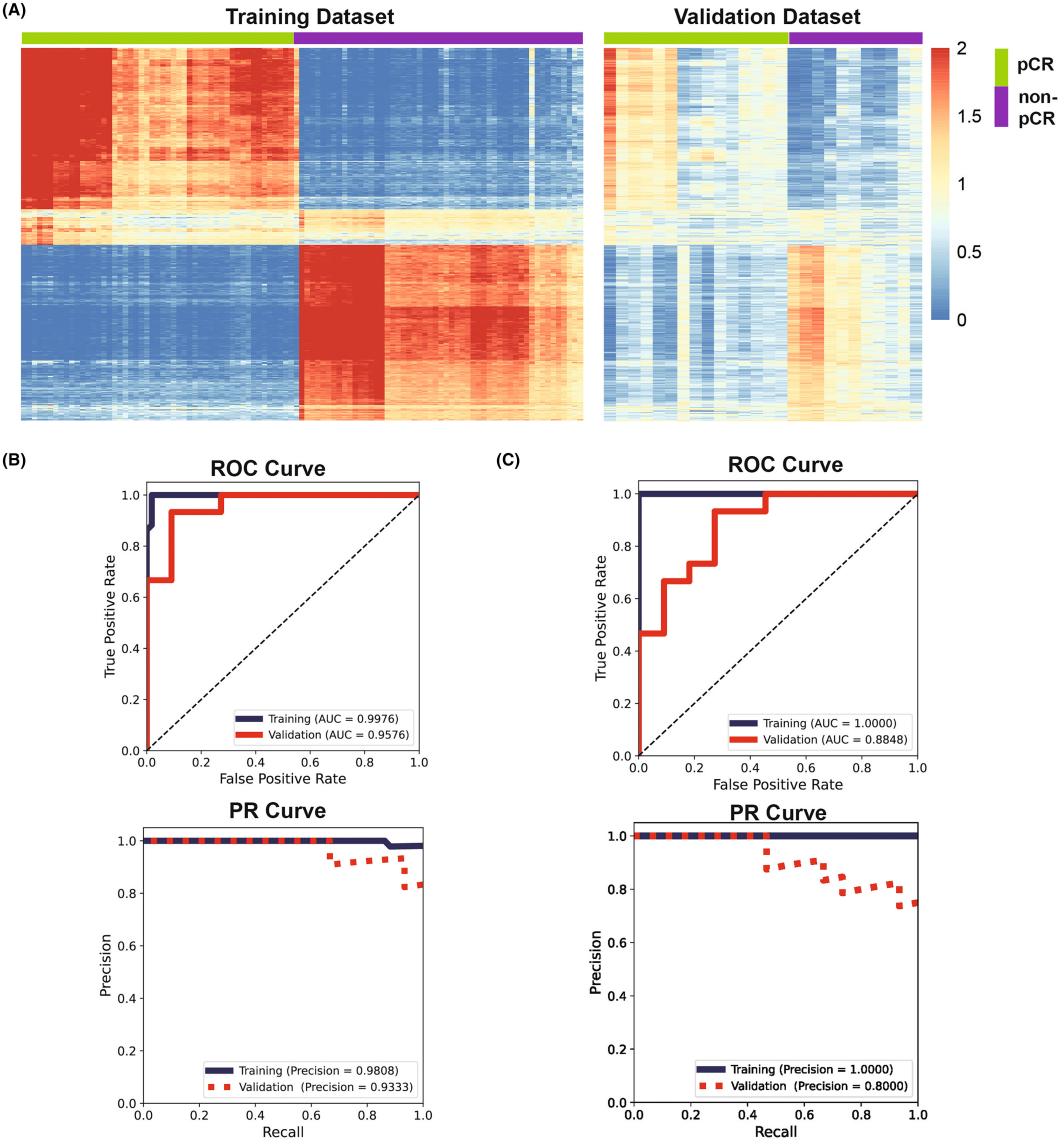
Deep learning signature (DLS) predicts pathologic complete response (pCR) to neoadjuvant chemotherapy. (A) Heatmap showing the quantitative values of 512 radiomic features forming the DLS. The 512 radiomic features were visualized in the radiogenomic training and radiogenomic validation dataset separately. Patients in the pCR and non‐pCR group were color‐coded. (B) Receiver operating characteristic curves (ROC) and progesterone receptor (PR) curves demonstrated the predictive power of the DLS in the radiogenomic training dataset (dark blue) and the radiogenomic validation dataset (red). (C) As in (B), but showing the ROC and PR curves for DLS trained by transfer learning.

A DLS was constructed from MR images to predict clinical outcomes following neoadjuvant chemotherapy. Deep features extracted from the fully connected layer of the network were visualized in the radiogenomic training and radiogenomic validation dataset (Data S2), which clearly separated the pCR and non‐pCR groups (Figure 3A). The DLS achieved the prediction accuracy, precision, recall, and F1 score of 0.923, 0.933, 0.933, and 0.933 in the radiogenomic validation dataset. The predictive power of the DLS was further assessed by ROC and PR (Figure 3B) curves. The DLS achieved AUC of 0.958 in the radiogenomic validation dataset. Furthermore, DCA curves were plotted to evaluate the clinical benefit of DLS prediction (Figure S1). The results demonstrated that administrating neoadjuvant chemotherapy following the prediction of DLS could bring significant clinical benefits comparing to treat all and treat none. Moreover, subtype analysis showed that the prediction accuracy of DLS was lower in HER2 and triple‐negative breast tumors compared to other molecular subtypes (Figure S2), but the AUC was above 0.92 for all subtypes.

Next, we sought to investigate if the application of transfer learning using a pretrained ResNet‐34 model could enhance the prediction accuracy. As depicted in Figure 3C, the DLS developed through transfer learning yielded a prediction accuracy, precision, recall, and F1 score of 0.77, 0.80, 0.80, and 0.80, respectively, in the radiogenomic validation dataset. This performance corresponded to an AUC of 0.88. These findings suggest that a ResNet‐34 model pretrained on ImageNet data is not effective in capturing MR features associated with clinical outcomes. Consequently, we decided to proceed with our investigation using the DLS that was trained on the radiogenomic training dataset.

### 3.3 PCR to neoadjuvant chemotherapy was underpinned by various biological pathways

**FIGURE 4:**
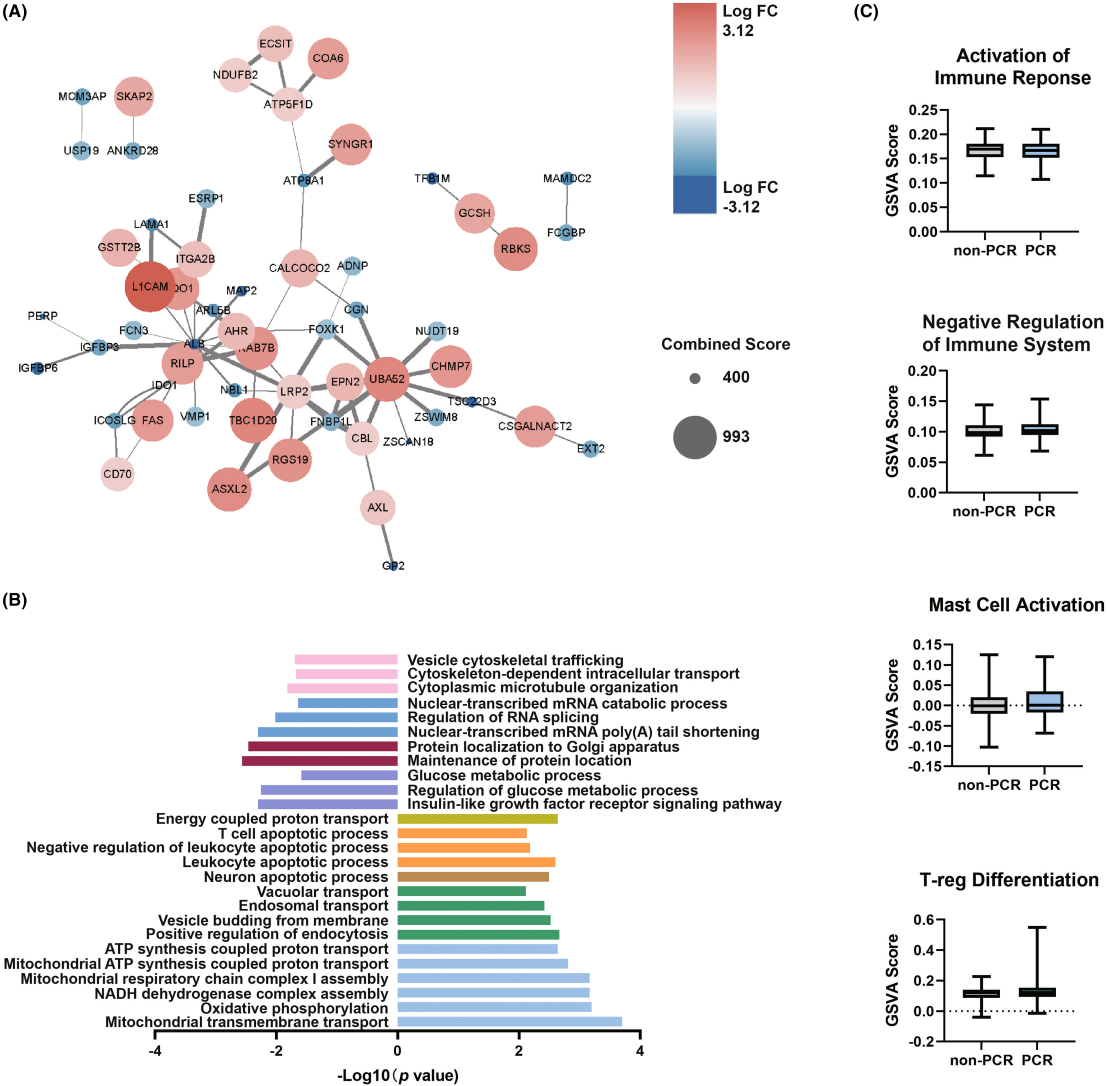
Deferentially expressed proteins (DEPs) and biological pathways upregulated in patients showing pathologic complete response (pCR) to neoadjuvant chemotherapy. (A) Protein–protein interaction (PPI) network of DEPs constructed using the STRING database. Connected nodes were visualized in Cytoscape, and node sizes were associated to the log fold change of the DEPs. Pink nodes were DEPs upregulated in the pCR group, and blue nodes denoted DEPs downregulated in the pCR group. (B) Enrichment analysis of the DEPs. The –log10 (*p*) values were visualized and color‐coded by the category of biological functions; light blue for mitochondrial energy metabolism, green for vesicle budding and vascular transportation, orange for leukocyte apoptotic process, purple for glucose metabolism, brick for protein localization to membranes, steel blue for messenger ribonucleic acid processing, and pink for cytoskeleton‐dependent trafficking. (C) Single‐sample GSEA scores of immune response and immune suppressive markers were shown by bloxplots. Patients showing PCR were indicated by gray boxes, whereas nonresponders were indicated by light blue boxes.

To investigate the biological meaning of the DLS, we first identified proteomic alterations associated with clinical responses to neoadjuvant chemotherapy. Liquid chromatography tandem mass spectrometry was performed on the pretreatment biopsy samples of 139 breast cancer patients. A total of 7842 proteins were quantified, among which 7055 proteins were present in more than 50% of the patients. The patients were divided into the pCR group and non‐PCR group depending on the treatment outcome of neoadjuvant chemotherapy. One hundred fifteen proteins were differentially expressed between the two groups (*p* < 0.05, FDR <0.05), including 55 upregulated proteins and 60 downregulated proteins in the pCR group. A PPI network was built with these DEPs (Figure 4A, combined score ≥400). Interestingly, the upregulated and downregulated DEPs did not form separate clusters. The majority of the hub genes of the PPI network were upregulated in the pCR group, including UBA52, LRP2, ATP5F1D, and RAB7B. The PPI network did not manifest canonical oncogenic pathways. Instead, the proteins on the network were primarily involved in cellular membrane formation, endocytosis, and insulin‐like growth factor binding.

To explore how these DEPs may underlie pCR to neoadjuvant chemotherapy, we conducted functional enrichment analysis on the upregulated and downregulated DEPs separately (Figure 4B). The pCR group upregulated DEPs were enriched for mitochondrial energy metabolism, vesicle budding and vascular transportation, and leukocyte apoptotic process. In contrast, the pCR group downregulated DEPs were enriched for glucose metabolism, protein localization to membranes, messenger ribonucleic acid (mRNA) processing, and cytoskeleton‐dependent trafficking. Next, we sought to investigate the difference among tumor molecular subtypes (Figure S3). Among patients showing PCR to neoadjuvant chemotherapy, triple‐negative breast tumors showed downregulated glucose metabolic process and higher maintenance of protein location. In nonresponders, triple‐negative breast tumors appeared to upregulate endosomal transport and downregulate leukocyte apoptosis. Furthermore, we sought to investigate whether nonresponders exhibited immunosuppression. Single‐sample enrichment scores of pathways including activation of immune response, negative regulation of immune system, mast cell activation, and Treg differentiation were compared between patients showing pCR and non‐PCR to neoadjuvant chemotherapy. However, no statistical difference was observed (Figure 4C).

### 3.4 Radiogenomic analysis revealed biological meanings of DLS

**FIGURE 5:**
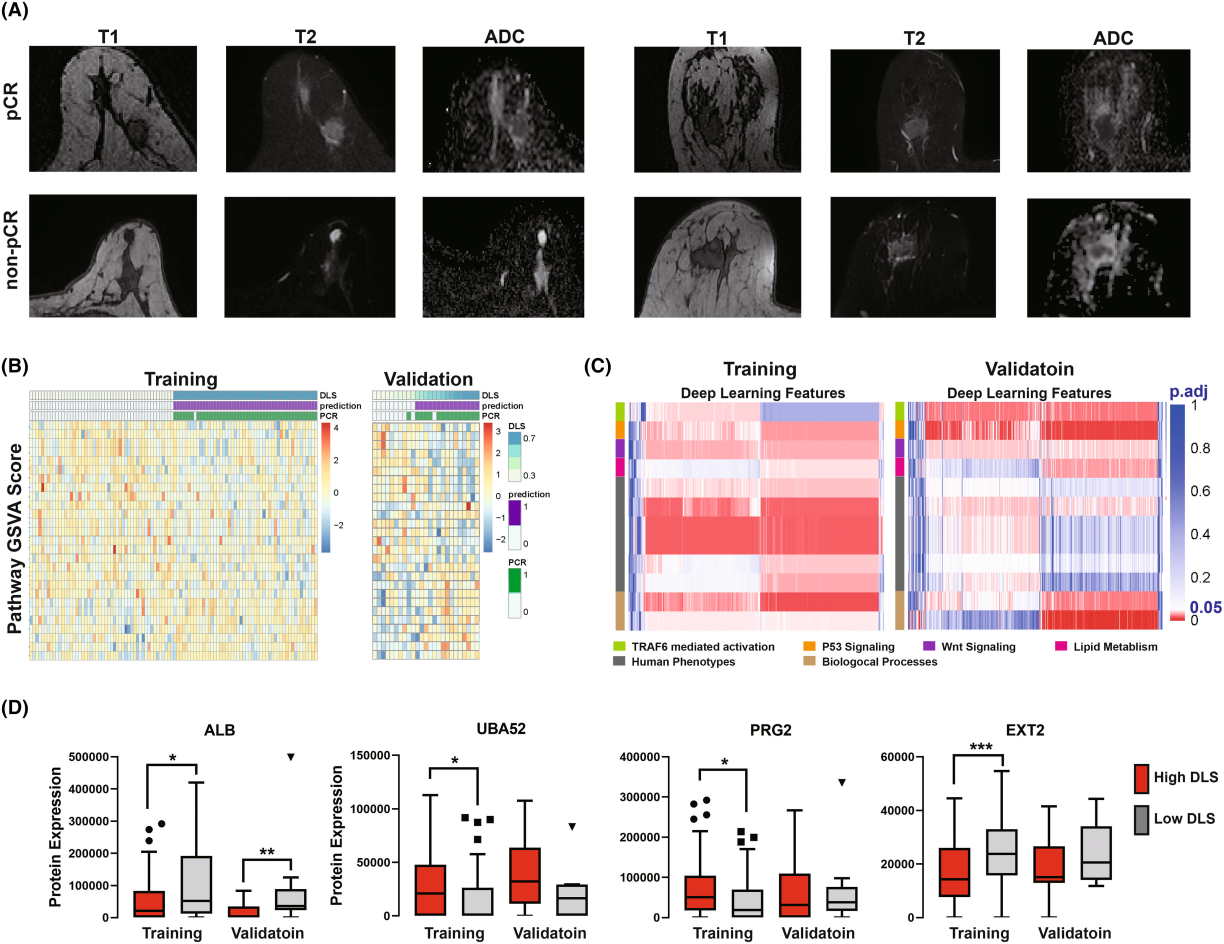
Deep learning signature (DLS) significantly correlated biological functions underlying pathologic complete response (pCR) to neoadjuvant chemotherapy. (A) Pretreatment MR images of tumors from the pCR (upper panel) and non‐pCR (lower panel) group. (B) Heatmap showing the gene set variation analysis scores of biological pathways significantly associated with DLS. (C) Heatmap showing correlations between 512 deep features and biological functions. Adjusted *p* values were indicated by color, *p*.adj < 0.05 was shown in red, and *p*.adj ≥ 0.05 was shown in blue. (D) Protein expression levels were compared between the high DLS (red) and low DLS (gray) groups in the radiogenomic training (*n* = 100) and validation (*n* = 25) datasets. *indicates *p* < 0.05, **indicates *p* < 0.01, ***indicates *p* < 0.001. Statistical difference was compared by Mann–Whitney test.

The pretreatment MR images of tumors showing pCR to neoadjuvant chemotherapy exhibited no morphological difference compared to the non‐responders (Figure 5A). Therefore, we sought to investigate the hidden association between DLS and the biological functions underlying pCR to neoadjuvant therapy. DLS was found to significantly correlate with GSVA scores of 1300 annotated biological pathways in radiogenomic training dataset, regardless of tumor molecular subtype. Associations with 27 pathways were confirmed in the radiogenomic validation dataset, including extracellular vesicle biogenesis, G protein‐coupled receptor internalization, microtubule end binding, adaptation of signaling pathway, and nuclear cyclin‐dependent protein kinase complex formation (Figure 5B). To illustrate the biological meaning of deep features forming the DLS, a heatmap was plotted to display the correlation between quantified deep features and core pathways as shown in Figure 5C. According to the results, the deep features can be categorized into three types: features correlated with oncogenic signaling pathways, features correlated with human phenotypes, and features correlated with general biological processes. Furthermore, the correlation between deep features and biological functions was identified in the radiogenomic training dataset and verified in the radiogenomic validation dataset.

By stratificating the patients using DLS, we showed that the expression of representative DEP such as ALB (albumin), UBA52 (ubiquitin carboxyl extension protein 52), PRG2 (proteoglycan 2), and EXT2 (exostosin glycosyltransferase 2) was significantly different in low DLS and high DLS groups (Figure 5D). In subtype analysis, the differences in ALB and EXT2 expression were also observed in luminal B and triple‐negative breast tumors, respectively (Figure S4). To conclude, the findings above conferred biological meanings to the DLS at multiple levels.

## 4 DISCUSSION

In this study, we established and validated a DLS using MRI to predict the treatment outcomes of neoadjuvant chemotherapy in breast cancer patients. Quantitative proteomic analysis was performed to explore the biological functions that facilitate pCR to neoadjuvant chemotherapy. The associations between the biological functions and the DLS were revealed by radiogenomic analysis. The main contribution of this study lies in two aspects: (1) We presented a DLS based on CNN networks to predict clinical outcomes of neoadjuvant chemotherapy noninvasively, and the DLS achieved the prediction AUC of 0.958 in the validation dataset; (2) pCR to neoadjuvant chemotherapy was underpinned by mitochondrial energy metabolism, protein localization to membranes, mRNA processing, and cytoskeleton‐dependent trafficking; these biological pathways were significantly associated with the DLS, which revealed the biological interpretability of the DLS.

Neoadjuvant chemotherapy is widely applied as the frontline therapy for the management of breast cancer. pCR to neoadjuvant chemotherapy is significantly correlated to better event‐free survival and overall survival.^31^ To select potential responders to neoadjuvant chemotherapy, biomarkers that could predict patient prognosis were extensively studied.^32^, ^33^, ^34^ Image features have emerged as a noninvasive tool for the prediction of patient prognosis. We have developed a DLS to predict pCR to neoadjuvant chemotherapy in breast cancer, achieving a prediction accuracy of 0.923 in the validation dataset. By extracting deep features from structural MRI, this non‐invasive approach allows the prediction of treatment outcomes before and during neoadjuvant chemotherapy and enables dynamic monitoring of the disease with minimal injury and financial cost.

Several studies have reported radiomic‐based machine learning models for predicting patient prognosis. Roy et al^35^ established a co‐clinical FDG‐PET radiomic signature from 131 radiomic features and achieved an accuracy of 78.48% to 86.21% in predicting response to neoadjuvant chemotherapy in triple‐negative breast cancer. MRI‐based radiomics extracted from microcalcification and peritumoral edema have also been applied to predict survival in breast cancer treated with neoadjuvant chemotherapy, showing a C‐index of 0.67–0.77 in the stratification of patient prognosis.^36^ According to a comparative study, the performance of radiomic‐based models, which employ MRI features, was superior to that of biologically based models using tumor cell features in predicting the response to neoadjuvant chemotherapy in triple‐negative breast cancer. The radiomic models achieved an impressive AUC of 0.89.^37^ Nonetheless, the majority of the studies have predominantly utilized handcrafted radiomic features as the input for their models. These handcrafted features are predefined and meticulously selected quantitative characteristics extracted from manually delineated tumor regions, intended to capture essential aspects of tumor morphology and texture. However, this approach has faced scrutiny due to its potential oversight of high‐order image features that lack a straightforward topological interpretation. Deep learning methods, on the other hand, allow for automated learning of latent features containing nonintuitive information. Previous studies have employed deep learning to improve medical imaging,^38^, ^39^ and preoperatively predict histologic grade in tumors.^40^ We constructed a DLS to predict pCR to neoadjuvant chemotherapy and achieved the prediction AUC of 0.958. Our result revealed the potential of DLS in extracting high‐order latent features from MR images, which facilitates improved model performance while boosting generalizability.

Our data also revealed that the precision accuracy of DLS vary among breast cancer subtypes, possibly due to the inherent biological and molecular heterogeneity of breast cancer. The molecular subtypes exhibit variations in gene expression patterns, signaling pathways, and response to treatments. Deep learning models rely on large datasets for training, and if the dataset used for training is predominantly composed of a specific subtype, the model may become biased toward recognizing patterns and features specific to that subtype. Consequently, when applied to other subtypes, the model's performance may decrease, leading to lower precision accuracy. To overcome this challenge, it is crucial to ensure diverse and representative datasets encompassing all breast cancer subtypes during the training phase, allowing the deep learning model to learn the unique features and patterns associated with each subtype, thereby improving its precision accuracy across all subtypes.

In contrast to handcrafted radiomic features, which are derived from well‐defined mathematical formulas, the interpretation of deep features learned through neural networks remains enigmatic. To shed light on the biological significance of the DLS, we performed a radiogenomic analysis to investigate the relationship between the DLS and the underlying biological functions associated with pCR to neoadjuvant chemotherapy. Consistent with previous publications, our data showed that mitochondrial energy metabolism, vesicle budding and vascular transportation, protein localization to membranes, mRNA processing, and cytoskeleton‐dependent trafficking were significantly altered in patients showing pCR to neoadjuvant chemotherapy.^41^ The enrichment score of these pathways significantly correlated with DLS, deep features forming DLS, and principle components of DLS, conferring biological interpretability to the predictive power of DLS at multiple levels.

Moreover, most of the previous studies tried to explore the biological meaning of deep features with transcriptomic data.^20^, ^21^ Transcriptomics measure rates of change due to ongoing transcription from mRNA, which is a proxy of protein concentration. However, proteomic data are the more reliable representation of the effector proteins because they are the end products of the transcription‐translation cascade that could reflect alterations caused by pre‐ and posttranslational modification. Therefore, our results based on proteomic analysis allowed apt characterization of the biological meaning of the DLS.

The major limitation of the study lies in the fact that the results lack external validation. Multicenter studies are required to confirm the predictive power and interpretability of the DLS. Furthermore, the sample size is relatively small due to the fact that paired proteomic data, MR images, and treatment response must be provided. The study should be repeated in a larger cohort to further validate our findings and train separate DLS models for each molecular subtype of breast cancer.

## 5 CONCLUSIONS

A DLS is established from pretreatment structural MR images, which was employed to predict treatment outcomes of neoadjuvant chemotherapy with high accuracy. The DLS significantly correlated with biological functions underpinning pCR to neoadjuvant chemotherapy, which provided the graphically obscure DLS with biological interpretability.

## AUTHOR CONTRIBUTIONS

**Jingxian Duan:** Conceptualization (equal); formal analysis (equal); funding acquisition (equal); methodology (equal); visualization (equal); writing – original draft (equal); writing – review and editing (equal). **Yuanshen Zhao:** Data curation (equal); investigation (equal); software (equal); validation (equal); visualization (equal); writing – original draft (equal). **Qiuchang Sun:** Resources (equal); software (equal); validation (equal); visualization (equal); writing – original draft (equal). **Dong Liang:** Project administration (equal); resources (equal); supervision (equal); writing – original draft (equal). **Zaiyi Liu:** Funding acquisition (equal); project administration (equal); resources (equal); supervision (equal); writing – review and editing (equal). **Xin Chen:** Conceptualization (equal); data curation (equal); funding acquisition (equal); project administration (equal); resources (equal); software (equal); supervision (equal); writing – review and editing (equal). **Zhi‐Cheng Li:** Conceptualization (equal); supervision (equal); writing – original draft (equal).

## FUNDING INFORMATION

This work was funded by the Key‐Area Research and Development Program of Guangdong Province, China (No. 2021B0101420006); National Natural Science Foundation of China (No. 62201557, U20A20171, 12126608, 82072090, and 61901458); Regional Innovation and Development Joint Fund of National Natural Science Foundation of China (No. U22A20345); Guangdong Provincial Key Laboratory of Artificial Intelligence in Medical Image Analysis and Application (No. 2022B1212010011); Guangdong Basic and Applied Basic Research Foundation (No. 2021A1515110585, 2020B1515120046); Science and technology Projects in Guangzhou (No.202201020001, 202201010513).

## CONFLICT OF INTEREST STATEMENT

The authors declare that they have no competing interests.

## ETHICS STATEMENT

The studies involving human participants were reviewed and approved by the Ethics Committee of Guangdong Provincial People's Hospital. Informed consent was obtained from all subjects involved in the study.

## Supporting information


Data S1.

Data S2.

Figures S1–S4.

## Data Availability

The datasets supporting the conclusions of this article are included within the article and its additional files.
