# Supplementary material for: Imaging‐proteomic analysis for prediction of neoadjuvant chemotherapy responses in patients with breast cancer
Source: Cancer Med. 2023 Nov 14;12(23):21256–69. doi: 10.1002/cam4.6704 (PMC10726892; doi:10.1002/cam4.6704)
Supplement: Supplementary file 3 — Figures S1–S4. [file CAM4-12-21256-s003.pdf]

Additional File 3-Supplementary Figures

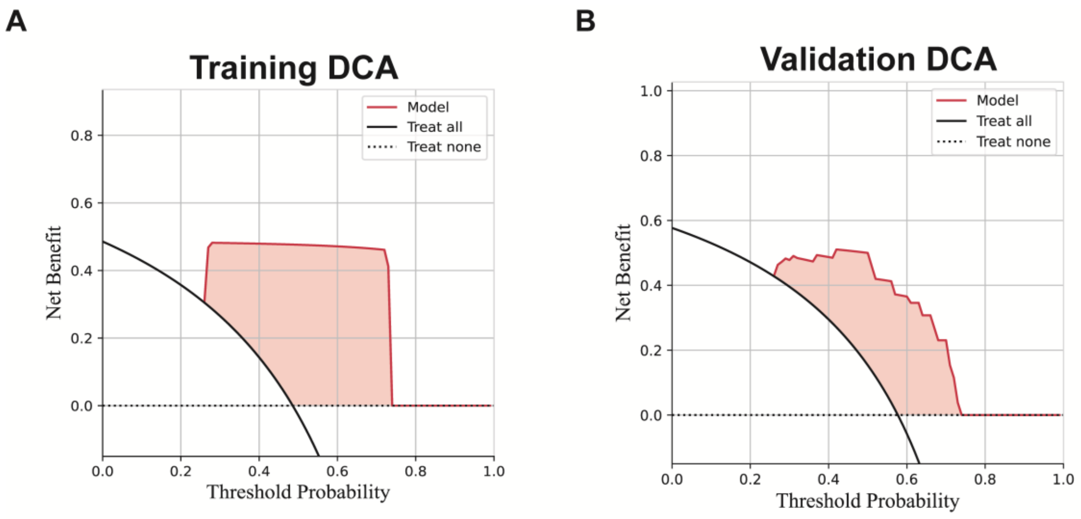

Supplementary Figure 1 DCA curves for the evaluation of the clinical benefit of DLS prediction

(A) Decision curve analysis of DLS in the radiogenomic training dataset. **(B)** As in (A), but for the validation dataset.

Subtype	Accuracy	Precision	Recall	F1	AUC
**HER2**	0.80	1.00	0.75	0.86	1.00
**Luminal A**	1.00	1.00	1.00	1.00	1.00
**Luminal B**	1.00	1.00	1.00	1.00	1.00
**Triple-negative**	0.90	0.83	1.00	0.91	0.92

(a)

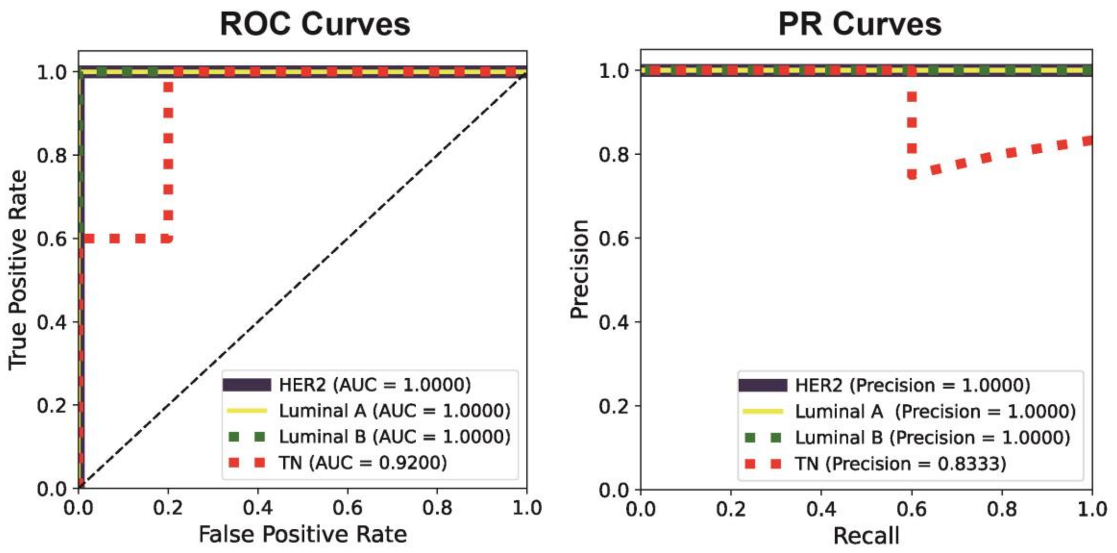

(b) (c)

Supplementary Figure 2 DLS predicts pCR to neoadjuvant chemotherapy in patients harboring tumors of different molecular subtypes.

(A) Table summarized accuracy, precision, recall F1 score, and AUC of DLS predicting pCR to neoadjuvant chemotherapy in patients harboring tumors of different molecular subtypes, including HER2, Luminal A, Luminal B, and Triple-negative (TN).. **(B)** ROC curve demonstrated the predictive power of the DLS in patients harboring tumors of different molecular subtypes. **(C)** As in (b), but showing the Precision-Recall (PR) curves.

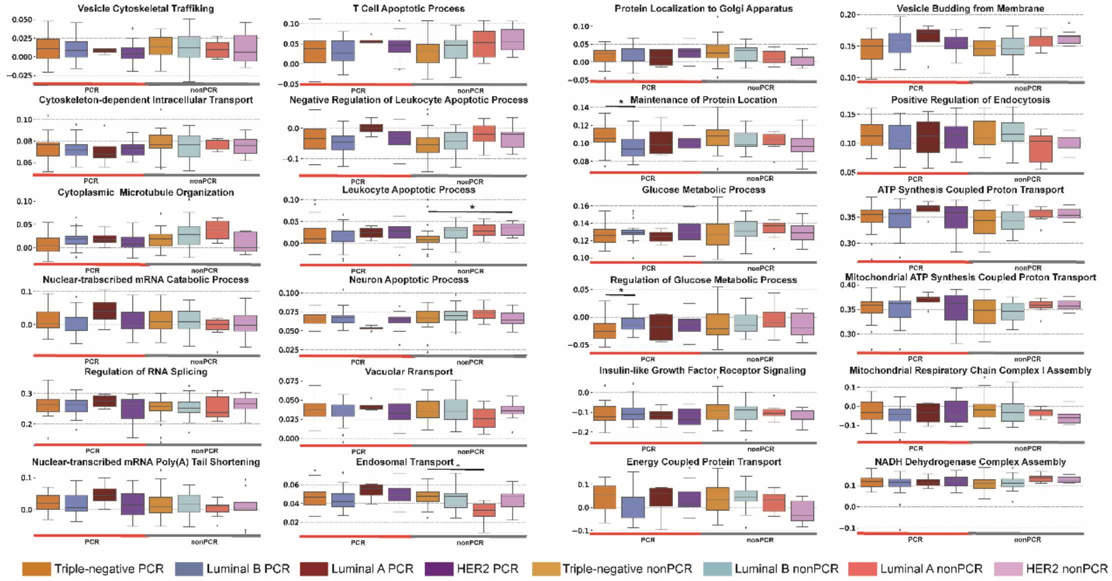

Supplementary Figure 3 Subtype analysis of significantly enriched pathways

The single-sample enrichment scores of significantly enriched pathways were calculated. The patients were divided into PCR and nonPCR groups based on their clinical outcomes. These patients were further sub-grouped by the molecular subtype of their tumors. The single-sample enrichment scores of patients of different sub-groups were compared. Statistical comparisons were conducted with unpaired t-test or Mann-Whitney test depending on the variation of the datasets. P values were calculated with the chi-square test. * for $p<0.05$, ** for $p<0.01$, *** for $p<0.001$, **** for $p<0.0001$.

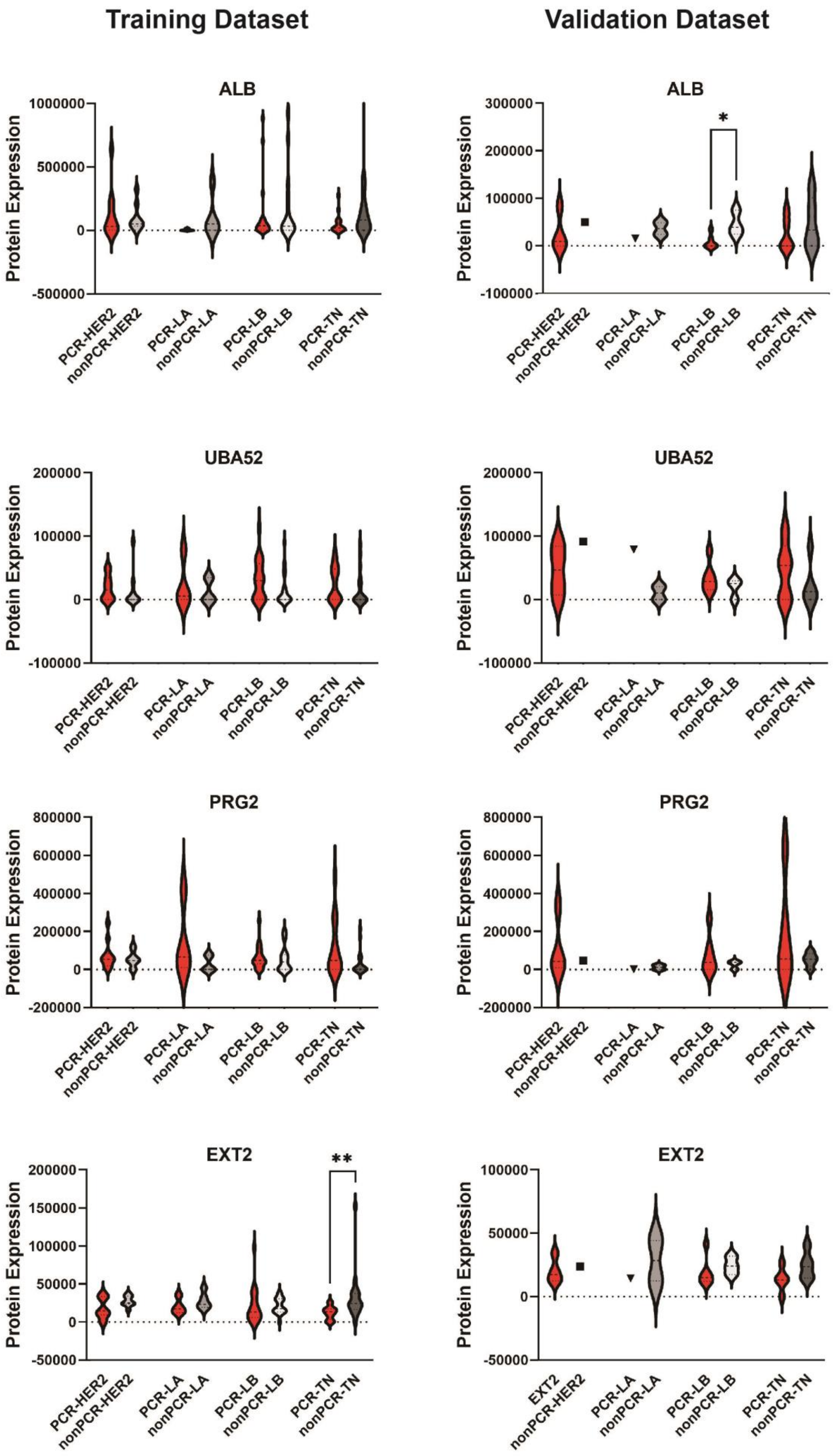
Training Dataset
Validation Dataset
ALB
UBA52
PRG2
EXT2
Protein Expression
1000000
500000
0
-500000
300000
200000
100000
-100000
50000
150000
-50000
800000
600000
400000
-200000
*
**
PCR-HER2
nonPCR-HER2
PCR-LA
nonPCR-LA
PCR-LB
nonPCR-LB
PCR-TN
nonPCR-TN
EXT2

Supplementary Figure 4 Expression of DEPs in tumor of different molecular subtypes

Protein expression of 4 representative DEPs were shown in the violin plots. Patients in training dataset and validation dataset were grouped by the molecular subtype of their tumors, including HER2, Luminal A (LA), Luminal B (LB), and Triple-negative (TN).Next, the patients were sub-grouped based on their clinical response to neoadjuvant chemotherapy. Protein expressions were compared across sub-groups. Statistical comparisons were conducted with unpaired t-test or Mann-Whitney test depending on the variation of the datasets. P values were calculated with the chi-square test. * for $p<0.05$, ** for $p<0.01$, *** for $p<0.001$, **** for $p<0.0001$.
